# Plasma B-type natriuretic peptide is independently associated with cardiovascular events and mortality in patients with chronic kidney disease

**DOI:** 10.1038/s41598-024-67529-1

**Published:** 2024-07-17

**Authors:** Hiroyuki Hayashida, Naoki Haruyama, Akiko Fukui, Ryota Yoshitomi, Hironobu Fujisawa, Masaru Nakayama

**Affiliations:** Division of Nephrology, Department of Internal Medicine, NHO Kyushu Medical Center, 1-8-1 Jigyohama, Chuo-ku, Fukuoka, 810-8563 Japan

**Keywords:** B-type natriuretic peptide, Chronic kidney disease, Cardiovascular event, Cardiac alteration, Medical research, Nephrology, Risk factors

## Abstract

The association between B-type natriuretic peptide (BNP) and cardiovascular (CV) events and mortality has not been well characterized in patients with chronic kidney disease (CKD). We prospectively investigated whether BNP was associated with CV events or mortality beyond cardiac alterations in 1078 patients with CKD. Participants were divided into the following 3 groups according to circulating BNP concentration: < 40 pg/mL, low; 40–100 pg/mL, middle; and > 100 pg/mL, high. Primary outcome was fatal or nonfatal CV events, and alternative outcome was a composite of fatal or nonfatal CV events, or non-CV deaths. During a median follow-up of 2.6 years, CV and composite events occurred in 158 and 248 participants, respectively. Cox analyses after adjustment for covariates, including cardiac parameters, showed that the hazard ratios (HRs) (95% confidence intervals [CIs]) for CV events of middle and high groups were 1.00 (0.63, 1.58) and 1.72 (1.06, 2.79), respectively, compared with low group. Additionally, similar results were obtained for composite events; the HRs (95% CIs) of middle and high groups were 1.10 (0.77, 1.57) and 1.54 (1.04, 2.27), respectively, compared with low group. Thus, in CKD, high BNP concentrations were independently associated with CV events and mortality, independent of cardiac alterations.

## Introduction

B-type natriuretic peptide (BNP) and N-terminal proBNP (NT-proBNP) are widely used in daily clinical practice as a marker of heart failure or volume overload. Myocytes in the left ventricle (LV) secrete pre-proBNP as a response to the stretching of myocytes by overload or volume-related expansion of the ventricle. Pre-proBNP is metabolized to proBNP, and proBNP is divided to BNP and NT-proBNP^1^. BNP is thought to bind to natriuretic peptide receptors and to be metabolized by enzymes such as neutral endopeptidase^2^, whereas NT-proBNP is thought to be principally excreted by the kidneys^3^. Additionally, a previous study demonstrated that kidney function contributed to concentrations of NT-proBNP, which predicted hemodynamic overload in patients with chronic kidney disease (CKD)^4^.

Cardiac natriuretic peptides, such as BNP and NT-proBNP, have been reported to be predictors for adverse outcomes. The associations of BNP and NT-proBNP with kidney disease progression have been documented in the general population^5^ and in patients with CKD^6–8^. Furthermore, high circulating BNP and/or NT-proBNP concentrations have been reported to be associated with cardiovascular (CV) events and mortality in patients with various cardiac diseases, including heart failure^9^, stable coronary artery disease^10^, and acute coronary syndrome^11^ and also in the general population^12^. In addition, several previous studies have demonstrated that high BNP^13–15^ and NT-proBNP concentrations^15–19^ are associated with substantial risks of CV events and mortality in patients with CKD.

A large left atrial diameter (LAD), a large left ventricular mass index (LVMI), and a low left ventricular ejection fraction (LVEF) have been reported to be associated with adverse CV outcomes in patients with CKD^20,21^. High BNP and/or NT-proBNP concentrations have also been shown to be associated with large LVMI, low LVEF, or large LAD in patients with CKD^14,15,22–24^. On the basis of these findings, cardiac parameters (e.g., LAD, LVMI, and LVEF) may be confounding factors in studies of the associations of these cardiac natriuretic peptides with CV events and mortality in patients with CKD. Therefore, the inclusion of these confounding variables as covariates should permit more accurate interpretations of the results of studies of these associations. To the best of our knowledge, a few studies have investigated the associations of NT-proBNP with CV events or mortality using multivariable Cox analyses that included cardiac parameters in patients with CKD^17,19^, but studies of the associations of BNP with CV events or mortality in such patients did not include cardiac parameters as covariates^13–15^. Therefore, in the present study, we aimed to determine whether circulating BNP concentration is associated with CV events and mortality in patients with CKD, independent of cardiac structure and function.

## Results

### Baseline clinical characteristics

The median age of the 1078 participants was 71 years (range 20–94 years) and their median estimated glomerular filtration rate (eGFR) was 30.3 mL/min/1.73 m^2^ (range 5.2–138.2 mL/min/1.73 m^2^). Of these, 190 (18%), 128 (12%), 225 (21%), 338 (31%), and 197 (18%) were categorized as having CKD stage G1–2, G3a, G3b, G4, and G5, respectively. Table 1 shows the baseline clinical characteristics of the participants. The participants with high BNP concentrations were older, more likely to be male, and more likely to have a history of smoking, diabetes mellitus, malignancy, and prior CV diseases (CVDs) such as ischemic heart disease (IHD), congestive heart failure (CHF), stroke, peripheral artery disease, aortic aneurysms, and aortic dissection. The number of participants who were taking renin–angiotensin–aldosterone system (RAAS) inhibitors, β-blockers, or diuretics increased with BNP concentration category. In addition, as BNP concentration increased, the systolic blood pressure, C-reactive protein (CRP), serum phosphorus, and proteinuria of the participants increased; and their body mass index (BMI), hemoglobin, serum albumin, and eGFR decreased. Furthermore, lower LVEF, larger LAD, and larger LVMI were associated with higher BNP concentrations.

**Table 1:** Baseline clinical characteristics of participants according to BNP levels.

Variables	All (*n* = 1078)	BNP (pg/mL)			*P* for trend
		Low (*n* = 573) (5.8–39.9 pg/mL)	Middle (*n* = 259) (40.2–99.6 pg/mL)	High (*n* = 246) (100.8–1431.5 pg/mL)	
Age (years)	71 (60, 78)	65 (50, 74)	75 (68, 80)	77 (70, 82)	< 0.01
Male,* n* (%)	695 (65)	355 (62)	160 (62)	180 (73)	< 0.01
Smoking status, *n* (%)	570 (53)	287 (50)	138 (53)	145 (59)	0.02
Diabetes mellitus,* n* (%)	380 (35)	163 (29)	108 (42)	109 (44)	< 0.01
Dyslipidemia, *n* (%)	772 (72)	420 (73)	195 (75)	157 (64)	0.02
Prior CVDs, *n* (%)	384 (36)	112 (20)	120 (46)	152 (62)	< 0.01
IHD, *n* (%)	148 (14)	30 (5)	56 (22)	62 (25)	< 0.01
CHF, *n* (%)	24 (2)	1 (0.2)	5 (2)	18 (7)	< 0.01
Stroke, *n* (%)	141 (13)	57 (10)	36 (14)	48 (20)	< 0.01
PAD, *n* (%)	155 (14)	43 (8)	48 (19)	64 (26)	< 0.01
Aortic aneurysm, *n* (%)	70 (6)	8 (1)	27 (10)	35 (14)	< 0.01
Aortic dissection, *n* (%)	8 (0.7)	0 (0)	4 (2)	4 (2)	< 0.01
Malignancy, *n* (%)	90 (8)	27 (5)	32 (12)	31 (13)	< 0.01
Use of RAAS inhibitor, *n* (%)	649 (60)	301 (53)	175 (68)	173 (70)	< 0.01
Use of β-blocker, *n* (%)	198 (18)	44 (8)	64 (25)	90 (37)	< 0.01
Use of diuretics, *n* (%)	291 (27)	91 (16)	88 (34)	112 (46)	< 0.01
Systolic blood pressure (mmHg)	132 (119, 144)	127 (117, 138)	134 (121, 148)	141 (129, 152)	< 0.01
Diastolic blood pressure (mmHg)	72 (65,80)	73 (67, 81)	69 (62, 77)	71 (63, 78)	< 0.01
BMI (kg/m^2^)	22.9 (20.5, 25.2)	23.2 (20.8, 26.0)	22.6 (20.2, 24.9)	21.9 (19.9, 24.1)	< 0.01
CRP (mg/L)	0.9 (0.5, 1.9)	0.8 (0.5, 1.7)	0.9 (0.5, 1.9)	1.2 (0.5, 2.5)	< 0.01
Hemoglobin (g/dL)	11.0 (9.5, 12.7)	12.0 (10.7, 13.5)	10.3 (9.3, 11.7)	9.5 (8.4, 10.9)	< 0.01
Seum albumin (g/dL)	3.5 (3.1, 3.8)	3.6 (3.3, 3.9)	3.4 (3.0, 3.7)	3.2 (2.8, 3.5)	< 0.01
Serum phosphorus (mg/dL)	3.7 (3.3, 4.1)	3.6 (3.3, 4.0)	3.6 (3.2, 4.1)	3.8 (3.3, 4.2)	< 0.01
eGFR (mL/min/1.73 m^2^)	30.3 (17.8, 49.8)	39.8 (23.8, 64.4)	25.7 (16.7, 37.7)	20.5(13.8, 31.7)	< 0.01
Daily proteinuria (g)	0.92 (0.22, 2.50)	0.75 (0.20, 1.90)	0.91 (0.22, 2.96)	1.51 (0.45, 3.23)	< 0.01
BNP (pg/mL)	34.5 (13.7, 88.4)	14.4 (6.9, 24.3)	60.9 (49.7, 75.5)	190.3 (132.8, 333.3)	< 0.01
LVEF (%)	69 (64, 74)	70 (66, 75)	70 (65, 75)	66 (60, 73)	< 0.01
LAD (mm)	39 (34, 44)	37 (32, 41)	40 (36, 45)	44 (38, 49)	< 0.01
LVMI (g/m^2^)	118 (94, 146)	103 (83, 127)	131 (107, 154)	147 (121, 182)	< 0.01

Values are expressed as number (percent) or median (interquartile range).

*BNP* B-type natriuretic peptide, *CVD* cardiovascular disease, *IHD* ischemic heart disease, *CHF* congestive heart failure, *PAD* peripheral artery disease, *RAAS* renin–angiotensin–aldosterone system, *BMI* body mass index, *CRP* C-reactive protein, *CRP* C-reactive protein, *eGFR* estimated glomerular filtration rate, *LVEF* left ventricular ejection fraction, *LAD* left atrial diameter, *LVMI* left ventricular mass index.

### Relationships between log BNP and other clinical parameters

Table 2 shows the variables that were found to be associated with log BNP using linear regression analysis. Simple analysis showed that log BNP was associated with many parameters, including demographics, smoking, diabetes mellitus, prior CV diseases, eGFR, LVEF, LAD, and LVMI. Multiple regression analysis showed that age, male sex, dyslipidemia, prior CV diseases, the use of β-blockers or diuretics, systolic blood pressure, BMI, hemoglobin concentration, serum albumin concentration, and parameters describing cardiac structure and function (LVEF, LAD, and LVMI), but not eGFR, were independent determinants of log BNP.

**Table 2:** Relationships between log BNP and other clinical parameters by linear regression analyses.

Variables	Simple linear regression analyses			Multiple linear regression analyses		
	Coefficients	95% CI	*P*	Coefficients	95% CI	*P*
Age (years)	0.042	(0.038, 0.047)	< 0.01	0.017	(0.012, 0.021)	< 0.01
Male	0.179	(0.020, 0.338)	0.03	− 0.142	(− 0.270, − 0.014)	0.03
Smoking	0.219	(0.067, 0.371)	0.01	− 0.005	(− 0.120, 0.110)	0.94
Diabetes mellitus	0.444	(0.287, 0.601)	< 0.01	− 0.351	(− 0.149, 0.079)	0.55
Dyslipidemia	− 0.127	(− 0.296, 0.042)	0.14	− 0.359	(− 0.473, − 0.244)	< 0.01
Prior CVDs	1.095	(0.950, 1.240)	< 0.01	0.304	(0.187, 0.422)	< 0.01
Malignancy	0.661	(0.388, 0.934)	< 0.01	0.095	(− 0.083, 0.273)	0.30
RAAS inhibitors	0.495	(0.342, 0.648)	< 0.01	− 0.100	(− 0.212, 0.012)	0.08
β-blockers	1.073	(0.887, 1.259)	< 0.01	0.527	(0.395, 0.660)	< 0.01
Diuretics	0.877	(0.714, 1.041)	< 0.01	0.196	(0.076, 0.315)	< 0.01
Systolic blood pressure (mm Hg)	0.024	(0.019, 0.028)	< 0.01	0.009	(0.005, 0.012)	< 0.01
BMI (kg/m^2^)	− 0.053	(− 0.072, − 0.034)	< 0.01	− 0.058	(− 0.073, − 0.043)	< 0.01
CRP (mg/L)	0.020	(0.005, 0.035)	0.01	0.002	(− 0.007, 0.012)	0.65
Hemoglobin (g/dL)	− 0.290	(− 0.320, − 0.261)	< 0.01	− 0.094	(− 0.126, − 0.001)	< 0.01
Serum albumin (g/dL)	− 0.680	(− 0.799, − 0.562)	< 0.01	− 0.294	(− 0.413, − 0.175)	< 0.01
Serum phosphorus (mg/dL)	0.180	(0.069, 0.290)	< 0.01	− 0.019	(− 0.100, 0.062)	0.64
eGFR (mL/min/1.73 m^2^)	− 0.022	(− 0.025, − 0.019)	< 0.01	− 0.002	(− 0.005, 0.001)	0.24
Daily proteinuria (g)	0.076	(0.047, 0.106)	< 0.01	0.012	(− 0.017, 0.041)	0.41
LVEF (%)	− 0.036	(− 0.045, − 0.028)	< 0.01	− 0.017	(− 0.022, − 0.011)	< 0.01
LAD (mm)	0.073	(0.064, 0.083)	< 0.01	0.034	(0.026, 0.043)	< 0.01
LVMI (g/m^2^)	0.015	(0.013, 0.016)	< 0.01	0.005	(0.004, 0.006)	< 0.01

*BNP* B-type natriuretic peptide, *CVD* cardiovascular disease, *RAAS* renin–angiotensin–aldosterone system, *BMI* body mass index, *CRP* C-reactive protein, *eGFR* estimated glomerular filtration rate, *LVEF* left ventricular ejection fraction, *LAD* left atrial diameter, *LVMI* left ventricular mass index.

### Associations of BNP with CV and composite events

**Figure 1:**
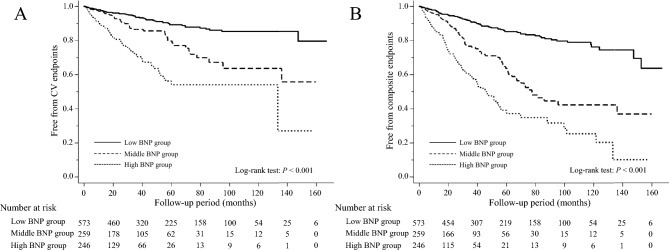
Kaplan–Meier curves for the absence of CV events (**A**) and the composite events (**B**) in participants stratified according to their BNP concentration, compared using the log-rank test. *CV* cardiovascular, *BNP* B-type natriuretic peptide.

During a median follow-up period of 2.6 years, fatal or nonfatal CV events and the composite events occurred in 148 and 248 participants, respectively. Total CV events comprised IHD in 34 participants, hospitalization for CHF in 42, stroke in 45, interventions for peripheral artery disease in 4, aortic dissection in 6, rupture of aortic aneurysms in 2, interventions for aneurysms of aorta or iliac artery in 12, interventions for internal carotid or vertebral artery stenosis in 2, cardiac valvular diseases in 3, and sudden death in 8. Kaplan–Meier analysis showed significantly higher prevalences of CV events (Fig. 1A) and the composite event (Fig. 1B) in patients with high BNP concentrations. Table 3 shows the hazard ratios (HRs) for CV and composite events. The fully adjusted Cox model showed that there was a significantly higher risk of CV events in the high BNP group compared with the low BNP group. High log BNP was also found to be independently associated with CV events. In addition, participants with high BNP concentrations had a significantly higher risk of the composite events compared with those with low BNP concentrations. Log BNP (a 1-log unit increase) was also independently associated with the composite events.

**Table 3:** Hazard ratios for CV and composite events of BNP levels.

	No. of events	Model 1		Model 2		Model 3		Model 4	
		HR (95% CI)	*P*	HR (95% CI)	*P*	HR (95% CI)	*P*	HR (95% CI)	*P*
CV events	158								
Low of BNP	52	Reference		Reference		Reference		Reference	
Middle of BNP	41	2.25 (1.49, 3.40)	< 0.01	1.31 (0.85, 2.02)	0.22	1.15 (0.74, 1.80)	0.53	1.00 (0.63, 1.58)	0.99
High of BNP	65	5.23 (3.60, 7.60)	< 0.01	2.96 (1.96, 4.46)	< 0.01	2.28 (1.46, 3.56)	< 0.01	1.72 (1.06, 2.79)	0.03
Log BNP (per 1-log unit increment)	–	1.81 (1.60, 2.04)	< 0.01	1.54 (1.33, 1.79)	< 0.01	1.41 (1.19, 1.66)	< 0.01	1.26 (1.05, 1.52)	0.02
Composite events	248								
Low of BNP	77	Reference		Reference		Reference		Reference	
Middle of BNP	74	2.96 (2.15, 4.08)	< 0.01	1.52 (1.09, 2.13)	0.01	1.22 (0.86, 1.72)	0.27	1.10 (0.77, 1.57)	0.62
High of BNP	97	6.01 (4.42, 8.17)	< 0.01	2.74 (1.96, 3.83)	< 0.01	1.84 (1.28, 2.63)	< 0.01	1.54 (1.04, 2.27)	0.03
Log BNP (per 1-log unit increment)	–	1.92 (1.74, 2.12)	< 0.01	1.52 (1.35, 1.71)	< 0.01	1.29 (1.13, 1.47)	< 0.01	1.21 (1.04, 1.40)	0.01

Model 1: Crude.

Model 2: Adjusted for age, sex, diabetes mellitus, dyslipidemia, smoking, systolic blood pressure, and BMI.

Model 3: Adjusted for model 2 plus prior CVDs, malignancy, CRP, hemoglobin, eGFR, and serum albumin.

Model 4: Adjusted for model 3 plus LVEF, LAD, and LVMI.

*CV* cardiovascular, *BNP* B-type natriuretic peptide, *HR* hazard ratio, *CI* confidence interval, *CVD* cardiovascular disease, *SBP* systolic blood pressure, *BMI* body mass index, *CRP* C-reactive protein, *eGFR* estimated glomerular filtration rate, *LVEF* left ventricular ejection fraction, *LAD* left atrial diameter, *LVMI* left ventricular mass index.

We also separated composite CV events into four categories: congestive heart failure (CHF), ischemic heart disease (IHD), stroke, and other CV events (i.e., peripheral artery disease, aortic dissection, rupture of aortic aneurysms, interventions for aneurysms of aorta or iliac artery, interventions for internal carotid or vertebral artery stenosis, cardiac valvular diseases, and sudden death). We investigated the associations of BNP with each CV endpoint. Supplementary Table 1 shows the HRs for each CV event of BNP levels. Multivariable Cox analyses found that participants with high BNP levels had a significant increase in the risk of CHF events, but not of IHD, stroke, or other CV events.

### Effects of BNP levels combined with prior CVD status on CV and composite events

Participants were also divided into the following six groups according to BNP levels and prior CVD status: low BNP and prior CVDs (−) (group A, *n* = 461), low BNP and prior CVDs (+) (group B, *n* = 112), middle BNP and prior CVDs (−) (group C, *n* = 139), middle BNP and prior CVDs (+) (group D, *n* = 120), high BNP and prior CVDs (−) (group E, *n* = 94), and high BNP and prior CVDs (+) (group F, *n* = 152). It was investigated whether the combination of high BNP levels and prior CVD status predicted the outcomes. In the fully adjusted Cox analyses, the HRs (95% CIs) for CV events in groups B through F were 2.01 (1.11, 3.66), 0.96 (0.48, 1.90), 1.90 (1.06, 3.40), 2.47 (1.26, 4.85), and 2.80 (1.54, 5.09), respectively, compared with group A. In the same model, the HRs (95% CIs) for composite events in groups B through F were 1.71 (1.05, 2.79), 1.24 (0.75, 2.04), 1.63 (1.02, 2.60), 1.80 (1.04, 3.13), and 2.29 (1.42, 3.70), respectively, compared with group A.

### Subgroup analyses regarding the associations of log BNP levels with CV and composite events

The adjusted HRs for CV events and the composite events per 1-log unit increment in log BNP in subgroups stratified by demographic and clinical characteristics are shown in Tables 4 and 5, respectively. No significant interactions for those outcomes were identified (*P* for interactions, 0.06–0.86).

**Table 4:** Adjusted hazard ratios for CV events per 1-log unit increment in log BNP among subgroups stratified by clinical parameters.

	No. of patients	No. of events	HR (95% CI)	*P*	*P* for interaction
Age					
Low (< 70.6 years)	539	48	1.35 (0.97, 1.89)	0.08	0.07
High (≥ 70.6 years)	539	110	1.18 (0.94, 1.48)	0.15	
Sex					
Male	695	114	1.32 (1.06, 1.63)	0.01	0.14
Female	383	44	1.44 (0.94, 2.19)	0.09	
Diabetes mellitus					
Absence	698	85	1.38 (1.05, 1.81)	0.02	0.06
Presence	380	73	1.16 (0.89, 1.52)	0.26	
Dyslipidemia					
Absence	306	36	1.60 (1.06, 2.41)	0.03	0.20
Presence	772	122	1.25 (1.01, 1.56)	0.04	
Prior CVDs					
Absence	694	63	1.36 (1.004, 1.85)	0.047	0.41
Presence	384	95	1.19 (0.94, 1.51)	0.14	
SBP					
Low (< 131.8 mmHg)	539	71	1.41 (1.05, 1.89)	0.02	0.23
High (≥ 131.8 mmHg)	539	87	1.20 (0.93, 1.55)	0.15	
BMI					
Low (< 22.9 kg/m^2^)	539	86	1.33 (1.04, 1.72)	0.03	0.45
High (≥ 22.9 kg/m^2^)	539	72	1.18 (0.89, 1.56)	0.26	
CRP					
Low (< 0.9 mg/L)	515	67	1.23 (0.91, 1.67)	0.18	0.82
High (≥ 0.9 mg/L)	563	91	1.27 (0.99, 1.63)	0.06	
Serum albumin					
Low (< 3.5 g/dL)	528	85	1.18 (0.92, 1.51)	0.19	0.39
High (≥ 3.5 g/dL)	550	73	1.35 (1.01, 1.81)	0.04	
eGFR					
Low (< 30.3 mL/min/1.73 m^2^)	539	90	1.20 (0.94, 1.54)	0.14	0.84
High (≥ 30.3 mL/min/1.73 m^2^)	539	68	1.24 (0.92, 1.69)	0.16	
LVEF					
Low (< 69.3%)	534	82	1.08 (0.84, 1.39)	0.53	0.28
High (≥ 69.3%)	544	76	1.52 (1.15, 2.02)	< 0.01	
LAD					
Low (< 39 mm)	534	49	1.09 (0.76, 1.56)	0.64	0.41
High (≥ 39 mm)	544	109	1.34 (1.08, 1.68)	0.01	
LVMI					
Low (< 118.0 g/m^2^)	539	50	1.43 (0.99, 2.08)	0.06	0.32
High (≥ 118.0 g/m^2^)	539	108	1.26 (1.00, 1.58)	0.049	

*CV* cardiovascular, *BNP* B-type natriuretic peptide, *HR* hazard ratio, *CI* confidence interval, *CVD* cardiovascular disease, *SBP* systolic blood pressure, *BMI* body mass index, *CRP* C-reactive protein, *eGFR* estimated glomerular filtration rate, *LVEF* left ventricular ejection fraction, *LAD* left atrial diameter, *LVMI* left ventricular mass index.

**Table 5:** Adjusted hazard ratios for composite events per 1-log unit increment in log BNP among subgroups stratified by clinical parameters.

	No. of patients	No. of events	HR (95% CI)	P	*P* for interaction
Age					
Low (< 70.6 years)	539	60	1.42 (1.05, 1.92)	0.02	0.06
High (≥ 70.6 years)	539	188	1.11 (0.94, 1.32)	0.23	
Sex					
Male	695	173	1.26 (1.06, 1.50)	0.01	0.09
Female	383	75	1.23 (0.90, 1.69)	0.19	
Diabetes mellitus					
Absence	698	142	1.36 (1.10, 1.68)	< 0.01	0.09
Presence	380	106	1.09 (0.88, 1.35)	0.44	
Dyslipidemia					
Absence	306	63	1.27 (0.93, 1.74)	0.13	0.41
Presence	772	185	1.20 (1.00, 1.42)	0.04	
Prior CVDs					
Absence	694	103	1.21 (0.94, 1.55)	0.14	0.37
Presence	384	145	1.18 (0.98, 1.42)	0.08	
SBP					
Low (< 131.8 mmHg)	539	116	1.35 (1.08, 1.69)	< 0.01	0.18
High (≥ 131.8 mmHg)	539	132	1.13 (0.92, 1.39)	0.25	
BMI					
Low (< 22.9 kg/m^2^)	539	145	1.28 (1.05, 1.56)	0.02	0.17
High (≥ 22.9 m^2^)	539	103	1.12 (0.88, 1.41)	0.36	
CRP					
Low (< 0.9 mg/L)	515	103	1.34 (1.05, 1.72)	0.02	0.43
High (≥ 0.9 mg/L)	563	145	1.14 (0.93, 1.38)	0.20	
Serum albumin					
Low (< 3.5 g/dL)	528	143	1.21 (0.99, 1.46)	0.06	0.62
High (≥ 3.5 g/dL)	550	105	1.28 (1.00, 1.64)	0.048	
eGFR					
Low (< 30.3 mL/min/1.73 m^2^)	539	151	1.10 (0.91, 1.32)	0.34	0.86
High (≥ 30.3 mL/min/1.73 m^2^)	539	97	1.31 (1.03, 1.67)	0.03	
LVEF					
Low (< 69.3%)	534	133	1.17 (0.95, 1.44)	0.14	0.80
High (≥ 69.3%)	544	115	1.27 (1.02, 1.59)	0.03	
LAD					
Low (< 39 mm)	534	87	1.04 (0.81, 1.34)	0.78	0.52
High (≥ 39 mm)	544	161	1.31 (1.09, 1.57)	< 0.01	
LVMI					
Low (< 118.0 g/m^2^)	539	80	1.27 (0.97, 1.67)	0.09	0.47
High (≥ 118.0 g/m^2^)	539	168	1.21 (0.999, 1.45)	0.05	

*CV* cardiovascular, *BNP* B-type natriuretic peptide, *HR* hazard ratio, *CI* confidence interval, *CVD* cardiovascular disease, *SBP* systolic blood pressure, *BMI* body mass index, *CRP* C-reactive protein, *eGFR* estimated glomerular filtration rate, *LVEF* left ventricular ejection fraction, *LAD* left atrial diameter, *LVMI* left ventricular mass index.

### Associations of BNP with CV and composite events in participants with LVEF ≥ 45% (sensitivity analyses)

We excluded 27 participants with LVEF < 45% who therefore may have had CHF, according to a previous study^25^. In the remaining 1051 participants, multivariable Cox analyses found that participants with high BNP levels had increased risk of both CV and composite events (Supplementary Table 2).

### Results of receiver operating characteristic (ROC) curve analyses for CV and composite events

The ROC curves for CV events (Supplementary Fig. 1) and composite events (Supplementary Fig. 2) were modelled according to stages 1–3 and 4–5 CKD. The area under the curve (AUC) for BNP with respect to CV events in participants with stages 1–3 and 4–5 CKD were 0.709 and 0.637, respectively (Supplementary Fig. 1A,B). AUC for BNP with respect to composite events in participants with stages 1–3 and 4–5 CKD were 0.733 and 0.640, respectively (Supplementary Fig. 2A,B). The cutoff values of BNP for CV events in participants with stages 1–3 and 4–5 CKD were 19.8 pg/mL (sensitivity = 0.786; specificity = 0.539) and 83.7 pg/mL (sensitivity = 0.591; specificity = 0.662), respectively. In addition, the cutoff values of BNP for composite events in participants with stages 1–3 and 4–5 CKD were 25.2 pg/mL (sensitivity = 0.727; specificity = 0.646) and 51.2 pg/mL (sensitivity = 0.711; specificity = 0.513), respectively.

### Baseline characteristics and HRs for CV and composite events of BNP levels after inverse probability of treatment weighting (IPTW)

Baseline characteristics after IPTW are shown in Supplementary Table 3. Additionally, the c-statistics between two groups based on propensity score were calculated as follows: low BNP *vs.* middle BNP, 0.904; low BNP *vs.* high BNP, 0.756; and middle BNP *vs.* high BNP, 0.747. Supplementary Table 4 shows the HRs for CV and composite events by BNP level after IPTW. Participants with high BNP levels had higher CV and composite events compared with those with low BNP levels. Additionally, log BNP levels were independently associated with both outcomes.

## Discussion

In the present study, we investigated whether high BNP concentrations are associated with CV events and mortality, independent of cardiac structure and function, in patients with CKD. Independent associations of log BNP with cardiac parameters (LAD, LVMI, and LVEF) were identified using multiple linear regression analysis, and even if these parameters were included in multivariable Cox analyses, high BNP concentration was identified as an independent risk factor for adverse outcomes (fatal or nonfatal CV events, and the composite event of fatal or nonfatal CV events, or non-CV death). Furthermore, subgroup analyses showed that baseline covariates such as prior CVDs, eGFR, LVEF, LAD, and LVMI did not significantly affect the associations between BNP and the two outcomes. Alternatively, we examined the effect of high BNP levels in combination with the prior presence of CVDs on poor outcomes. It was confirmed that the combination had strongest predictive power for adverse events. Furthermore, the associations of higher BNP levels with adverse outcomes in multivariable analyses after IPTW were similar to the findings obtained before IPTW.

In the heart–kidney hemodynamic model, the kidneys control the extracellular fluid volume by regulating sodium excretion and reabsorption, and the heart controls systemic hemodynamics. When one of these organs fails, a vicious circle develops in which the RAAS, the nitric oxide-reactive oxygen species balance, the sympathetic nervous system, and inflammation interact and synergize, thereby leading to an increase in morbidity and mortality^26,27^. Cardiorenal syndrome (CRS) is defined as a pathophysiologic disorder of the heart and kidneys, whereby acute or chronic dysfunction in one organ induces acute or chronic dysfunction in the other. Five subtypes of this syndrome have also been defined: type 1, acute cardio-renal; type 2, chronic cardio-renal; type 3, acute reno-cardiac; type 4, chronic reno-cardiac; and type 5, secondary CRS. In CRS type 2, chronic cardiac abnormalities result in kidney injury or dysfunction. In contrast, in CRS type 4, CKD contributes to a decrease in cardiac function, cardiac hypertrophy, and/or an increase in the risk of adverse CV events^28,29^. To date, several studies have demonstrated that poor kidney function or a decline in function are associated with higher risks of CVD and mortality^30–32^. Cardiac biomarkers, such as BNP and NT-proBNP, have shown prognostic value in patients at various stages of kidney dysfunction, and therefore have potential applications in patients with CRS types 2 or 4^28^.

Several previous studies have documented associations of BNP/NT-proBNP with kidney function or cardiac alterations. A previous study demonstrated that BNP concentrations are less affected than those of NT-proBNP by kidney dysfunction^33^. Vickery et al*.* reported that LVMI is independently associated with both BNP and NT-proBNP, whereas eGFR had an independent effect on the BNP, and especially on the NT-proBNP, concentrations in patients with CKD^24^. BNP concentration has also been reported to be independently associated with left ventricular overload, but not with kidney function^14^. Furthermore, Tagore et al*.* reported that there is an independent inverse correlation between eGFR and NT-proBNP, but not BNP, in patients with stages 3 or 4 CKD^23^. In the present study, simple linear regression analysis revealed a significant inverse association between log BNP and eGFR, but multiple linear regression analysis did not. In addition, in multiple analysis, independent associations between log BNP and cardiac parameters were identified, as in previous studies^24^. These findings suggest that in patients with CKD, BNP concentration may be influenced by abnormalities in cardiac structure and function, rather than kidney dysfunction. However, the previous studies that characterized the associations of BNP with CV outcomes and mortality in patients with CKD did not include cardiac parameters as covariates^13–15^; therefore, the fact that significant associations of BNP concentrations with adverse outcomes identified in the present study were independent of indices of cardiac structure and function provides valuable additional information.

Several previous studies have addressed a variety of CV endpoints: all-cause deaths^9,11,12,15^, CV deaths^9–11^, CHF^9–15^, IHD including myocardial infarction^9–13,15^, stroke^10–13,15^, atrial fibrillation^12^, aortic dissection^13^, and aortic aneurysm rupture^13^. Among these, three large cohort studies^10–12^ have reported the associations between BNP and category of CV event. In all three studies, BNP was associated with CHF. Conversely, the associations of BNP with atherosclerotic CV events (e.g., IHD and stroke) were inconsistent. A significant association between BNP and stroke was found in one study^12^, but not in the other two studies^10,11^. All three studies reported no evidence of association between BNP and IHD including myocardial infarction^10–12^. In the present study, a robust association between BNP and CHF events was confirmed, while no significant associations between BNP, and IHD and stroke were observed.

Volume overload is not uncommon in patients with CKD and is associated with both traditional and nontraditional CV risk factors^34^. A previous study demonstrated that volume overload, assessed using bioelectric impedance spectroscopy, is an independent risk factor for CV morbidity and all-cause mortality in patients with advanced CKD stages^35^. Furthermore, a previous systematic review demonstrated that volume overload was associated with CHF events^36^. In the present study, the volume status of the participants was not assessed using a body composition monitor, but their LADs were assessed using echocardiography, and log BNP was found to be independently and positively associated with LAD. It has previously been reported that LAD has a significant positive correlation with estimated plasma volume^37^, and BNP has been reported to be a biomarker of volume status in patients with CKD^38^. Therefore, in the present cohort, participants with high BNP concentrations may have had more severe volume overload. Furthermore, the present study found a significant association of BNP with CHF events, consistent with other several studies^10–12,14^. Given these findings, the implied association between high BNP and volume overload might explain the link between high BNP concentration and adverse outcomes, especially CHF events.

BNP concentration may increase in response to myocardial ischemia, even if this is not accompanied by heart failure^39,40^. Therefore, it may have value for the identification of subclinical cardiac disease in patients who do not have clinical heart failure^41^. Subclinical myocardial injury has also been reported to be associated with higher risks of CVD and all-cause mortality in individuals who do not have CVD^42^. In a previous study, BNP was also reported to be associated with nonfatal myocardial infarction in patients with stable coronary heart disease^43^. Therefore, high BNP-related subclinical myocardial injury may be explained by the association between BNP and IHD events. However, like previous large cohort studies^10–12^, the present study showed no significant association between BNP and IHD events. Furthermore, findings on the association of BNP and stroke are also conflicting. A previous large cohort study^12^ reported a significant association between BNP and stroke; conversely, two large cohort studies^10,11^ and the present study did not. Given these findings, it remains unclear whether higher BNP levels may contribute to subsequent CV events such as IHD or stroke, and further investigations are warranted to determine the association of BNP with these CV events.

The present study had several limitations. First, all the participants were recruited at a single regional hospital; therefore, the sample was fairly homogeneous and subject to selection bias. Second, we recruited consecutive patients who were admitted to the hospital; they were relatively old, were all Japanese, and the number of male participants was approximately 1.8-times higher than that of female participants. Third, we did not measure the concentrations of cardiac troponin I (cTnI) which is a sensitive and specific marker of myocardial injury^44^. Higher cTnI was also reported to be associated with a large spectrum of incident CV events in the community-based cohort^45^. Furthermore, it was demonstrated that combined measurements of cTnI and BNP were more reliable predictors of increased CV events in patients with hypertrophic cardiomyopathy^46^. Therefore, simultaneous measurements of these cardiac biomarkers could provide more novel information regarding the risk stratification for subsequent CV events. Fourth, we did not evaluate the New York Heart Association function class for each participant. We performed sensitivity analyses in participants with LVEF ≥ 45%; however, it remained unclear whether these participants did or did not have CHF. Fifth, the numbers of CV events by category (i.e., CHF, IHD, stroke, and other CV events) were relatively small. Therefore, statistical power may have been low when performing multivariable Cox analyses. Sixth, although the maximum absolute standardized difference (ASD) of each covariate after IPTW was lower compared with that calculated before applying IPTW, maximum ASDs in all covariates after IPTW were not less than 0.1 (as shown in Supplementary Table 5). An ASD of < 0.1 has been taken to indicate a negligible difference in the mean or prevalence of a covariate between treatment groups^47^. Therefore, the patient population after IPTW may not be well-balanced across all covariates. In clinical epidemiology, a very high c-statistic value can indicate considerable non-overlap in propensity score distribution between exposed and unexposed subjects^48^. Furthermore, it is possible that IPTW may perform poorly when the treatment groups are initially very different and when some patients have extreme propensity scores near 1 or 0^49^. In the present study, the c-statistics were also calculated between each two-group comparison. In particular, the c-statistic between the low and middle BNP groups was > 0.9, suggesting very high non-overlap between the two groups. Accordingly, this may indicate unstable estimation via IPTW. Finally, we only used a single BNP measurement, which may not be a highly accurate predictor of adverse outcomes. In optimally treated patients with chronic heart failure, the use of a BNP-guided strategy has been reported to be associated with a lower risk of chronic heart failure-related death or hospitalization because of chronic heart failure than the use of a conventional strategy based on clinical expertise^50^. In addition, a recent study demonstrated that BNP monitoring is associated with a lower risk of a requirement for kidney replacement therapy in patients with CKD who are not on dialysis^51^. Therefore, further studies are warranted to determine whether longitudinal BNP monitoring lower the risks of CV events and mortality in patients with CKD.

In conclusion, in patients with CKD, high BNP concentrations are associated with adverse outcomes, such as CV events and mortality, independent of confounding variables such as cardiac parameters. These findings suggest that BNP is a useful biomarker of the risk associated with adverse outcomes in patients with CKD.

## Methods

### Study design

Between June 2009 and November 2022, 1279 consecutive Japanese patients who were admitted to the NHO Kyushu Medical Center for the evaluation of, and education regarding, CKD were selected. Of these, we excluded 61 who showed acute-on-chronic kidney injury, 7 who lacked BNP data, and 5 who did not undergo echocardiography. The remaining 1206 patients were discharged from hospital without initiating kidney replacement therapy and were subsequently followed up at the same hospital. Of these, 60 who were lost to follow-up within 6 months of discharge and 68 who initiated kidney replacement therapy within the same period were also excluded. Therefore, data for 1078 patients that were collected up to June 2023 were prospectively analyzed.

The study was approved by the Ethics Committee of the NHO Kyushu Medical Center (approval number: 09-09), registered with the University Hospital Medical Information Network (UMIN000017519), and performed in accordance with the guidelines of the Declaration of Helsinki. Written informed consent was obtained from all the participants.

### Outcome definitions

The primary endpoints were fatal or nonfatal CV events in the absence of kidney replacement therapy, which were defined as follows: IHD (those requiring percutaneous intervention or coronary artery bypass grafting, acute myocardial infarction, and myocardial ischemia identified using myocardial scintigraphy), hospitalization for the treatment of CHF, stroke (brain infarction, brain hemorrhage, subarachnoid hemorrhage, and non-traumatic acute subdural hematoma), requirement for interventions to treat peripheral artery disease (percutaneous transcatheter angioplasty, lower-limb amputation, and bypass surgery), dissecting aneurysm of the thoracic and/or abdominal aorta, rupture of a thoracic and/or abdominal aortic aneurysm, requirement for bypass or stent placement in the thoracic or abdominal aortic aneurysm, or iliac artery aneurysm, requirement for stent placement for the treatment of internal carotid or vertebral artery stenosis, cardiac valvular diseases (sudden onset of severe aortic regurgitation, valve replacement surgery, or transcatheter aortic valve implantation for the treatment of aortic stenosis), and sudden death. The alternative outcome was a composite of fatal or nonfatal CV events, or non-CV deaths, without the initiation of kidney replacement therapy. Non-CV death was defined as death that occurred in the absence of a CV event. The follow-up period was defined as the period between baseline and a first event in participants who experienced events, or as the time to the completion of the study or loss to follow-up in participants who were censored.

### Data collection

Blood samples (serum creatinine, serum phosphorus, hemoglobin, serum albumin, CRP, and BNP) were obtained from participants early in the morning following an overnight fast on the second day of admission. Daily proteinuria was also measured. eGFR (mL/min/1.73 m^2^) was calculated using the following new Japanese equation: eGFR = 194 × SCr^−1.094^ × age^−0.287^ (× 0.739 if female), where SCr is the serum creatinine concentration of the participant^52^.

All the participants were interviewed regarding their medical history, including hypertension, diabetes mellitus, prior CVDs, and malignancy. The demographic information (age and sex), medication history, and history of smoking at presentation were recorded for each participant. Prior CVDs were defined as a history of IHD, CHF, stroke, peripheral artery disease, thoracic and/or abdominal aortic aneurysm, and/or aortic dissection. Peripheral artery disease was defined as having a low ankle–brachial blood pressure index (< 0.9) or having undergone treatment for lower limb ischemia. Participants were also categorized according to their cigarette smoking status as current or past smokers. BMI was calculated as body mass in kg divided by height in m, squared. Blood pressure was measured on three separate occasions on day 2 of hospitalization, with the participants in a sitting position, and the mean of the three values obtained was recorded.

### Assessment of cardiac structure and function

Left ventricular mass (LVM) was calculated using M-mode data obtained from parasternal long-axis images, according to the following formula^53^: LVM = 1.04 ([IVSd + LVPWd + LVDd]^3^ − LVDd^3^) − 13.6, where IVSd and LVPWd are the thicknesses of the interventricular septum and the posterior wall of the LV during diastole, respectively, and LVDd is the diameter of the LV during diastole. LVMI was expressed as LVM per square meter of body surface area, calculated using the Du Bois formula^54^ as body mass^0.425^ × height^0.725^ × 0.007184.

### Statistical analyses

Continuous data are expressed as the median (interquartile range) because Shapiro–Wilk testing indicated that none of the continuous variables were normally distributed. The participants were divided into the following three categories based on their BNP concentration, according to the JCS 2017/JHFS 2017 Guidelines on Diagnosis and Treatment of Acute and Chronic Heart Failure^55^: BNP levels < 40 pg/mL, low group; 40–100 pg/mL, middle group; and > 100 pg/mL, high group. The BNP concentration was non-normally distributed; therefore, it was log-transformed to achieve an approximately normal distribution prior to statistical analysis. Linear regression analysis was performed to identify variables associated with log BNP. Multiple linear regression analyses were adjusted for all variables used in simple linear regression analyses to determine independent variables for log BNP. Survival curves were constructed using the Kaplan–Meier method and evaluated using the log-rank test. We selected traditional CV risk factors (age, sex, smoking, diabetes mellitus, systolic blood pressure, dyslipidemia, BMI, and prior CVDs); non-traditional CV risk factors (hemoglobin, CRP, serum albumin, and eGFR); the presence of malignancy, which affects mortality; and cardiac parameters (LAD, LVEF, and LVMI, which are closely related to BNP concentration) as covariates. Common variables associated with both univariable linear regression for log BNP and Cox analyses for outcomes were considered confounding factors, and the above covariates—with the exceptions of dyslipidemia, malignancy, daily proteinuria, and BMI—were confirmed to be confounding factors. Cox proportional hazards models were used to determine whether BNP was associated with CV and the composite events, and HRs and 95% confidence intervals were calculated for each variable. Subgroup analyses were performed according to sex, the presence or absence of categorical variables, and the status of continuous data (values below or above the median value). The effects of interactions between log BNP and other variables on the outcomes were evaluated by adding interaction terms for the associations between log BNP and other variables to the relevant model. ROC curve analyses were used to evaluate the diagnostic performance of BNP for the prediction of CV and composite events, and the sensitivity and specificity of BNP to predict CV and composite events were also determined, according to stages 1–3 and 4–5 CKD. The propensity scores were evaluated using multivariable logistic regression models that included the following variables: age, sex, smoking, diabetes mellitus, dyslipidemia, prior CVDs, malignancy, systolic blood pressure, BMI, CRP, hemoglobin, serum albumin, eGFR, LVEF, LAD, and LVMI. Inverse probability of treatment weighting (IPTW) was calculated to balance the covariates among three groups, namely: low, middle, and high BNP groups^56^. We used Stata’s *streg* command, which fits proportional hazard models with a Weibull distribution, to evaluate the association of BNP levels with CV and composite events^57^. Statistical analyses were performed using STATA version 15 (Stata Corp., College Station, TX, USA), and *P* < 0.05 was taken to indicate statistical significance.

### Supplementary Information

Supplementary Table 1.

Supplementary Table 2.

Supplementary Table 3.

Supplementary Table 4.

Supplementary Table 5.

Supplementary Figure 1.

Supplementary Figure 2.

## Data Availability

The datasets generated and/or analyzed during the present study will be made available by the corresponding author upon reasonable request.
