# Supplementary material for: Plasma B-type natriuretic peptide is independently associated with cardiovascular events and mortality in patients with chronic kidney disease
Source: Sci Rep. 2024 Jul 17;14:16542. doi: 10.1038/s41598-024-67529-1 (PMC11255297; doi:10.1038/s41598-024-67529-1)
Supplement: Supplementary file 1 — Supplementary Table 1. [file 41598_2024_67529_MOESM1_ESM.docx]

**Supplementary Table1** Hazard ratios for each CV event of BNP levels

|  |  | No. of events | Model 1 | | Model 2 | | Model 3 | | Model 4 | |
| --- | --- | --- | --- | --- | --- | --- | --- | --- | --- | --- |
|  |  |  | HR (95% CI) | *P* | HR (95% CI) | *P* | HR (95% CI) | *P* | HR (95% CI) | *P* |
| CHF | | 42 |  |  |  |  |  |  |  |  |
|  | Low of BNP | 6 | reference | | reference | | reference | | reference | |
|  | Middle of BNP | 10 | 4.73 (1.72, 13.0) | <0.01 | 2.80 (0.98, 8.02) | 0.06 | 2.60 (0.88, 7.65) | 0.08 | 1.78 (0.60, 5.33) | 0.30 |
|  | High of BNP | 26 | 18.9 (7.69, 46.5) | <0.01 | 10.8 (4.04, 28.7) | <0.01 | 9.57 (3.33, 27.5) | <0.01 | 4.84 (1.65, 14.2) | <0.01 |
|  | Log BNP (per 1-log unit increment) | – | 2.55 (2.02, 3.21) | <0.01 | 2.36 (1.77, 3.14) | <0.01 | 2.31 (1.68, 3.18) | <0.01 | 1.75 (1.25, 2.45) | <0.01 |
| IHD | | 34 |  |  |  |  |  |  |  |  |
|  | Low of BNP | 19 | reference | | reference | | reference | | reference | |
|  | Middle of BNP | 8 | 1.18 (0.51, 2.70) | 0.70 | 0.62 (0.26, 1.47) | 0.28 | 0.51 (0.20, 1.26) | 0.14 | 0.54 (0.21, 1.38) | 0.20 |
|  | High of BNP | 7 | 1.42 (0.59, 3.42) | 0.43 | 0.90 (0.35, 2.31) | 0.83 | 0.77 (0.28, 2.09) | 0.60 | 0.91 (0.31, 2.69) | 0.87 |
|  | Log BNP (per 1-log unit increment) | – | 1.23 (0.94, 1.62) | 0.13 | 0.98 (0.69, 1.38) | 0.89 | 0.85 (0.58, 1.24) | 0.40 | 0.96 (0.63, 1.48) | 0.87 |
| Stroke | | 45 |  |  |  |  |  |  |  |  |
|  | Low of BNP | 17 | reference | | reference | | reference | | reference | |
|  | Middle of BNP | 13 | 2.30 (1.11, 4.74) | 0.03 | 1.44 (0.67, 3.08) | 0.34 | 1.30 (0.58, 2.88) | 0.52 | 1.27 (0.56, 2.90) | 0.57 |
|  | High of BNP | 15 | 4.12 (2.02, 8.40) | <0.01 | 2.21 (1.02, 4.82) | 0.045 | 1.84 (0.79, 4.26) | 0.16 | 1.74 (0.69, 4.41) | 0.24 |
|  | Log BNP (per 1-log unit increment) | – | 1.60 (1.27, 2.01) | <0.01 | 1.27 (0.96, 1.67) | 0.10 | 1.16 (0.86, 1.58) | 0.32 | 1.14 (0.80, 1.62) | 0.46 |
| Other CV events | | 37 |  |  |  |  |  |  |  |  |
|  | Low of BNP | 10 | reference | | reference | | reference | | reference | |
|  | Middle of BNP | 10 | 2.73 (1.13, 6.58) | 0.03 | 1.59 (0.63, 4.05) | 0.33 | 1.35 (0.52, 3.54) | 0.54 | 1.27 (0.47, 3.45) | 0.64 |
|  | High of BNP | 17 | 6.38 (2.87, 14.14) | <0.01 | 3.90 (1.62, 9.39) | <0.01 | 2.48 (0.97, 6.36) | 0.06 | 2.12 (0.75, 5.95) | 0.16 |
|  | Log BNP (per 1-log unit increment) | – | 1.96 (1.53, 2.51) | <0.01 | 1.80 (1.33, 2.44) | <0.01 | 1.50 (1.08, 2.08) | 0.02 | 1.42 (0.95, 2.11) | 0.08 |

Model 1: Crude.

Model 2: Adjusted for age, sex, diabetes mellitus, dyslipidemia, smoking, systolic blood pressure, and BMI.

Model 3: Adjusted for model 2 plus prior CVDs, malignancy, CRP, hemoglobin, eGFR, and serum albumin.

Model 4: Adjusted for model 3 plus LVEF, LAD, and LVMI.

Abbreviations: CHF, congestive heart failure; IHD, ischemic heart disease; CV, cardiovascular; BNP, B-type natriuretic peptide; HR, hazard ratio; CI, confidence interval; BMI, body mass index; CVD, cardiovascular disease; CRP, C-reactive protein; eGFR, estimated glomerular filtration rate; LVEF, left ventricular ejection fraction; LAD, left atrial diameter; LVMI, left ventricular mass index.
