# Supplementary material for: Plasma B-type natriuretic peptide is independently associated with cardiovascular events and mortality in patients with chronic kidney disease
Source: Sci Rep. 2024 Jul 17;14:16542. doi: 10.1038/s41598-024-67529-1 (PMC11255297; doi:10.1038/s41598-024-67529-1)
Supplement: Supplementary file 2 — Supplementary Table 2. [file 41598_2024_67529_MOESM2_ESM.docx]

**Supplementary Table 2** Hazard ratios for CV and composite events of BNP levels in participants with preserved LVEF (LVEF ≥ 45%)

|  |  | No. of events | Model 1 | | Model 2 | | Model 3 | | Model 4 | |
| --- | --- | --- | --- | --- | --- | --- | --- | --- | --- | --- |
|  |  |  | HR (95% CI) | *P* | HR (95% CI) | *P* | HR (95% CI) | *P* | HR (95% CI) | *P* |
| CV events | | 146 |  |  |  |  |  |  |  |  |
|  | Low of BNP | 52 | reference | | reference | | reference | | reference | |
|  | Middle of BNP | 40 | 2.25 (1.49, 3.41) | <0.01 | 1.31 (0.85, 2.02) | 0.23 | 1.16 (0.74, 1.83) | 0.51 | 1.06 (0.66, 1.69) | 0.81 |
|  | High of BNP | 54 | 4.75 (3.21, 7.01) | <0.01 | 2.70 (1.75, 4.20) | <0.01 | 2.13 (1.33, 3.41) | <0.01 | 1.74 (1.04, 2.91) | 0.04 |
|  | Log BNP (per 1-log unit increment) | – | 1.75 (1.54, 1.98) | <0.01 | 1.47 (1.26, 1.73) | <0.01 | 1.32 (1.11, 1.57) | <0.01 | 1.26 (1.03, 1.53) | 0.03 |
| Composite events | | 236 |  |  |  |  |  |  |  |  |
|  | Low of BNP | 77 | reference | | reference | | reference | | reference | |
|  | Middle of BNP | 73 | 3.00 (2.17, 4.14) | <0.01 | 1.54 (1.10, 2.17) | 0.01 | 1.23 (0.87, 1.75) | 0.24 | 1.15 (0.80, 1.65) | 0.45 |
|  | High of BNP | 86 | 5.83 (4.25, 8.00) | <0.01 | 2.68 (1.89, 3.81) | <0.01 | 1.83 (1.26, 2.66) | <0.01 | 1.63 (1.09, 2.44) | 0.02 |
|  | Log BNP (per 1-log unit increment) | – | 1.91 (1.72, 2.11) | <0.01 | 1.50 (1.32, 1.70) | <0.01 | 1.26 (1.10, 1.45) | <0.01 | 1.22 (1.05, 1.42) | 0.01 |

Model 1: Crude.

Model 2: Adjusted for age, sex, diabetes mellitus, dyslipidemia, smoking, systolic blood pressure, and BMI.

Model 3: Adjusted for model 2 plus prior CVDs, malignancy, CRP, hemoglobin, eGFR, and serum albumin.

Model 4: Adjusted for model 3 plus LVEF, LAD, and LVMI.

Abbreviations: CV, cardiovascular; BNP, B-type natriuretic peptide; HR, hazard ratio; CI, confidence interval; BMI, body mass index; CVD, cardiovascular disease; CRP, C-reactive protein; eGFR, estimated glomerular filtration rate; LVEF, left ventricular ejection fraction; LAD, left atrial diameter; LVMI, left ventricular mass index.
