# Supplementary material for: Plasma B-type natriuretic peptide is independently associated with cardiovascular events and mortality in patients with chronic kidney disease
Source: Sci Rep. 2024 Jul 17;14:16542. doi: 10.1038/s41598-024-67529-1 (PMC11255297; doi:10.1038/s41598-024-67529-1)
Supplement: Supplementary file 3 — Supplementary Table 3. [file 41598_2024_67529_MOESM3_ESM.docx]

**Supplementary Table 3** Baseline clinical characteristics of participants according to BNP levels after IPTW

| Variables |  | BNP (pg/mL) | | |
| --- | --- | --- | --- | --- |
|  | All (*n* = 2,924) | Low (*n* = 1,025)  (5.8–39.9 pg/mL) | Middle (*n* = 1,058)  (40.2–99.6 pg/mL) | High (*n* = 840)  (100.8–1431.5 pg/mL) |
| Age (years) | 73 (62, 79) | 70 (60, 78) | 71 (60, 78) | 74 (66, 81) |
| Male, *n* (%) | 1,884 (64) | 657 (64) | 637 (60) | 591 (70) |
| Smoking status, *n* (%) | 1,497 (51) | 558 (54) | 515 (49) | 425 (51) |
| Diabetes mellitus, *n* (%) | 1,095 (37) | 370 (36) | 398 (38) | 327 (39) |
| Dyslipidemia, *n* (%) | 2,046 (70) | 740 (72) | 736 (70) | 569 (68) |
| Prior CVDs, *n* (%) | 1,088 (37) | 340 (33) | 383 (36) | 365 (43) |
| Malignancy, *n* (%) | 284 (10) | 101 (10) | 86 (8) | 96 (11) |
| Systolic blood pressure (mmHg) | 132 (118, 144) | 130 (119, 141) | 133 (118, 145) | 132 (116, 146) |
| BMI (kg/m²) | 22.6 (20.4, 24.9) | 22.9 (20.7, 24.9) | 23.2 (20.4, 25.3) | 21.9 (19.6, 24.3) |
| CRP (mg/dL) | 0.09 (0.05, 0.18) | 0.09 (0.05, 0.17) | 0.09 (0.05, 0.18) | 0.10 (0.05, 0.18) |
| Hemoglobin (g/dL) | 10.9 (9.4, 12.6) | 11.1 (9.8, 12.7) | 10.8 (9.5, 12.3) | 10.6 (8.9, 12.4) |
| Seum albumin (g/dL) | 3.4 (3.1, 3.7) | 3.5 (3.2, 3.8) | 3.5 (3.1, 3.9) | 3.4 (3.0, 3.5) |
| eGFR (mL/min/1.73 m²) | 29.3 (17.6, 45.9) | 29.5 (17.8, 51.0) | 31.9 (18.2, 45.2) | 27.7 (15.9, 40.0) |
| BNP (pg/mL) | 56.1 (28.7, 107) | 19.0 (10.5, 28.9) | 60.6 (50.4, 70.3) | 150 (112, 220) |
| LVEF (%) | 69 (65, 75) | 69 (65, 74) | 70 (65, 75) | 68 (64, 75) |
| LAD (mm) | 39 (34, 44) | 38 (34, 43) | 39 (34, 44) | 40 (35, 44) |
| LVMI (g/m²) | 120 (96, 149) | 117 (93, 147) | 119 (94, 146) | 125 (102, 161) |

Values are expressed as number (percent) or median (interquartile range).

Abbreviations: BNP, B-type natriuretic peptide; IPTW, inverse probability of treatment weighting; CVD, cardiovascular disease; BMI, body mass index; CRP, C-reactive protein; eGFR, estimated glomerular filtration rate; LVEF, left ventricular ejection fraction; LAD, left atrial diameter; LVMI, left ventricular mass index.
