# Supplementary material for: Plasma B-type natriuretic peptide is independently associated with cardiovascular events and mortality in patients with chronic kidney disease
Source: Sci Rep. 2024 Jul 17;14:16542. doi: 10.1038/s41598-024-67529-1 (PMC11255297; doi:10.1038/s41598-024-67529-1)
Supplement: Supplementary file 4 — Supplementary Table 4. [file 41598_2024_67529_MOESM4_ESM.docx]

**Supplementary Table 4** Hazard ratios for CV and composite events of BNP levels after IPTW

|  |  | No. of events | Model 1 | | Model 2 | | Model 3 | | Model 4 | |
| --- | --- | --- | --- | --- | --- | --- | --- | --- | --- | --- |
|  |  |  | HR (95% CI) | *P* | HR (95% CI) | *P* | HR (95% CI) | *P* | HR (95% CI) | *P* |
| CV events | | 491 |  |  |  |  |  |  |  |  |
|  | Low of BNP | 121 | reference | | reference | | reference | | reference | |
|  | Middle of BNP | 149 | 1.19 (0.70, 2.02) | 0.52 | 1.19 (0.72, 1.96) | 0.50 | 1.16 (0.70, 1.92) | 0.58 | 1.12 (0.67, 1.90) | 0.66 |
|  | High of BNP | 222 | 2.83 (1.41, 5.71) | <0.01 | 2.67 (1.40, 5.11) | <0.01 | 2.44 (1.23, 4.85) | 0.01 | 2.36 (1.18, 4.71) | 0.02 |
|  | Log BNP (per 1-log unit increment) | – | 1.75 (1.41, 2.17) | <0.01 | 1.63 (1.26, 2.10) | <0.01 | 1.52 (1.15, 2.00) | <0.01 | 1.47 (1.10, 1.95) | 0.01 |
| Composite events | | 809 |  |  |  |  |  |  |  |  |
|  | Low of BNP | 222 | reference | | reference | | reference | | reference | |
|  | Middle of BNP | 253 | 1.10 (0.68, 1.79) | 0.70 | 1.06 (0.71, 1.59) | 0.78 | 1.04 (0.70, 1.55) | 0.83 | 1.06 (0.71, 1.57) | 0.79 |
|  | High of BNP | 334 | 2.42 (1.47, 3.96) | <0.01 | 1.96 (1.11, 3.47) | 0.02 | 1.80 (1.01, 3.23) | 0.047 | 1.83 (1.04, 3.22) | 0.04 |
|  | Log BNP (per 1-log unit increment) | – | 1.66 (1.39, 1.99) | <0.01 | 1.44 (1.15, 1.80) | <0.01 | 1.31 (1.04, 1.66) | 0.02 | 1.30 (1.04, 1.63) | 0.02 |

Model 1: Crude.

Model 2: Adjusted for age, sex, diabetes mellitus, dyslipidemia, smoking, systolic blood pressure, and BMI.

Model 3: Adjusted for model 2 plus prior CVDs, malignancy, CRP, hemoglobin, eGFR, and serum albumin.

Model 4: Adjusted for model 3 plus LVEF, LAD, and LVMI.

Abbreviations: CV, cardiovascular; BNP, B-type natriuretic peptide; IPTW, inverse probability of treatment weighting; HR, hazard ratio; CI, confidence interval; BMI, body mass index; CVD, cardiovascular disease; CRP, C-reactive protein; eGFR, estimated glomerular filtration rate; LVEF, left ventricular ejection fraction; LAD, left atrial diameter; LVMI, left ventricular mass index.
