# Supplementary material for: Plasma B-type natriuretic peptide is independently associated with cardiovascular events and mortality in patients with chronic kidney disease
Source: Sci Rep. 2024 Jul 17;14:16542. doi: 10.1038/s41598-024-67529-1 (PMC11255297; doi:10.1038/s41598-024-67529-1)
Supplement: Supplementary file 5 — Supplementary Table 5. [file 41598_2024_67529_MOESM5_ESM.docx]

**Supplementary Table 5** ASDs across all two-group comparisons before and after IPTW

|  | Low BNP *vs.* Middle BNP | | Low BNP *vs.* High BNP | | Middle BNP *vs.* High BNP | | Maximum ASDs | |
| --- | --- | --- | --- | --- | --- | --- | --- | --- |
| Variables | before | after | before | after | before | after | before | after |
| Age | 0.908 | 0.018 | ***1.067*** | 0.387 | 0.192 | ***0.485*** | 1.067 | 0.485 |
| Gender | 0.004 | 0.081 | 0.241 | 0.134 | ***0.245*** | ***0.218*** | 0.245 | 0.218 |
| Smoking status | 0.064 | ***0.115*** | ***0.178*** | 0.077 | 0.114 | 0.038 | 0.178 | 0.115 |
| Diabetes mellitus | 0.280 | 0.031 | ***0.334*** | ***0.059*** | 0.053 | 0.027 | 0.334 | 0.059 |
| Dyslipidemia | 0.046 | 0.060 | 0.205 | ***0.098*** | ***0.251*** | 0.041 | 0.251 | 0.098 |
| Prior CVDs | 0.594 | 0.068 | ***0.951*** | ***0.231*** | 0.313 | 0.146 | 0.951 | 0.231 |
| Malignancy | 0.276 | 0.062 | ***0.283*** | 0.057 | 0.007 | ***0.099*** | 0.283 | 0.099 |
| Systolic blood pressure | 0.393 | 0.091 | ***0.712*** | ***0.119*** | 0.316 | 0.030 | 0.712 | 0.119 |
| BMI | 0.235 | 0.046 | ***0.355*** | ***0.254*** | 0.124 | 0.215 | 0.355 | 0.254 |
| CRP | 0.084 | ***0.064*** | ***0.186*** | 0.050 | 0.117 | 0.006 | 0.186 | 0.064 |
| Hemoglobin | 0.802 | 0.145 | ***1.087*** | ***0.231*** | 0.319 | 0.094 | 1.087 | 0.231 |
| Serum albumin | 0.441 | 0.111 | ***0.714*** | ***0.344*** | 0.208 | 0.201 | 0.714 | 0.344 |
| eGFR | 0.637 | 0.026 | ***0.992*** | 0.313 | 0.347 | ***0.411*** | 0.992 | 0.411 |
| LVEF | 0.181 | 0.029 | ***0.535*** | ***0.173*** | 0.349 | 0.135 | 0.535 | 0.173 |
| LAD | 0.538 | 0.108 | ***1.016*** | ***0.213*** | 0.509 | 0.111 | 1.016 | 0.213 |
| LVMI | 0.725 | 0.114 | ***1.162*** | ***0.292*** | 0.487 | 0.178 | 1.162 | 0.292 |

Italic numerals with bold indicate maximum ASDs before and after IPTW.

Abbreviations: ASD, absolute standardized differences; IPTW, inverse probability of treatment weighting; BNP, B-type natriuretic peptide; CVD, cardiovascular disease; BMI, body mass index; CRP, C-reactive protein; eGFR, estimated glomerular filtration rate; LVEF, left ventricular ejection fraction; LAD, left atrial diameter; LVMI, left ventricular mass index.
