# Supplementary material for: Plasma B-type natriuretic peptide is independently associated with cardiovascular events and mortality in patients with chronic kidney disease
Source: Sci Rep. 2024 Jul 17;14:16542. doi: 10.1038/s41598-024-67529-1 (PMC11255297; doi:10.1038/s41598-024-67529-1)
Supplement: Supplementary file 7 — Supplementary Figure 2. [file 41598_2024_67529_MOESM7_ESM.pptx]

## Slide 1
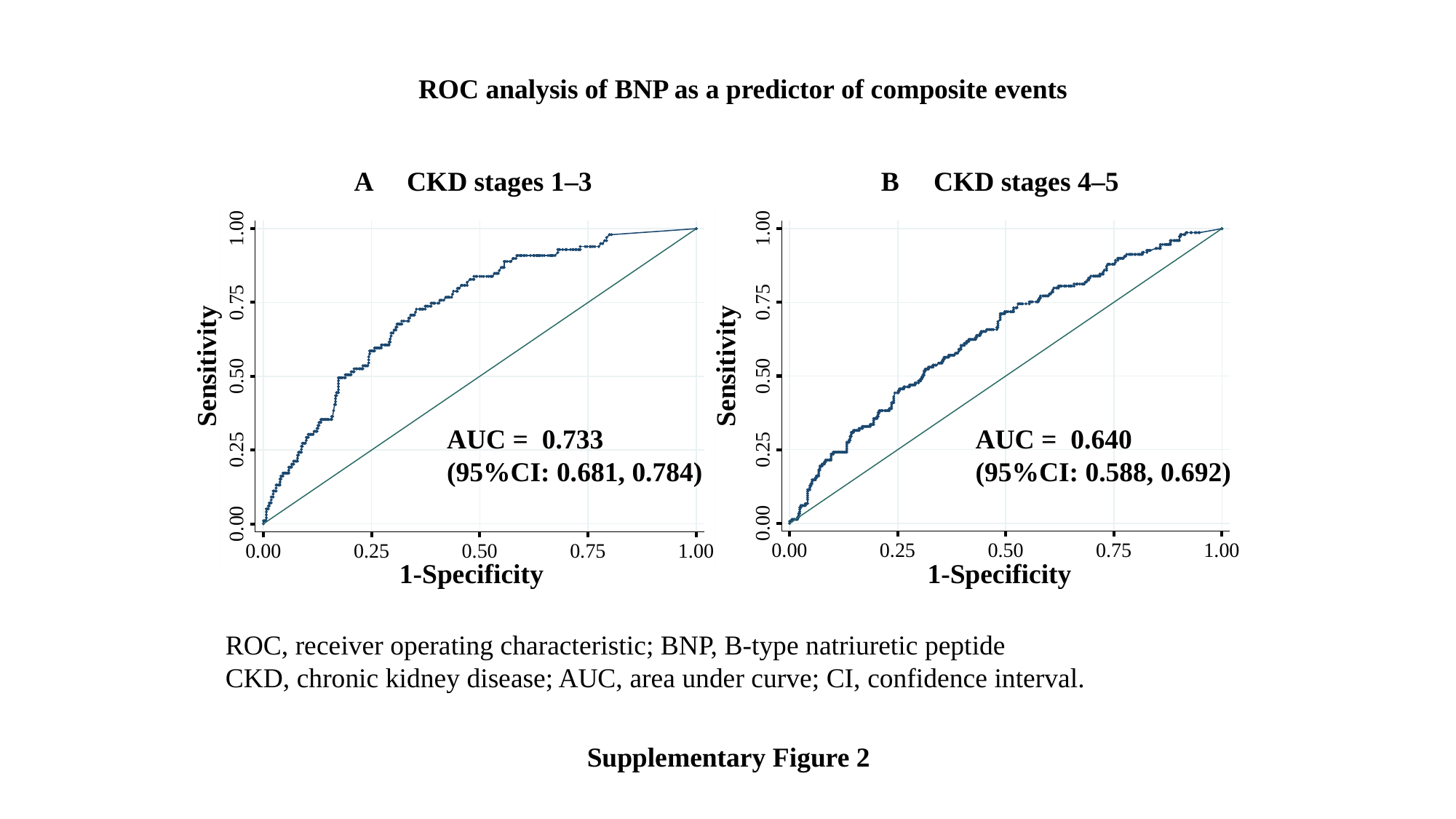

ROC analysis of BNP as a predictor of composite events
A CKD stages 1–3
B CKD stages 4–5
Sensitivity
Sensitivity
AUC = 0.733
(95%CI: 0.681, 0.784)
AUC = 0.640
(95%CI: 0.588, 0.692)
1-Specificity
1-Specificity
ROC, receiver operating characteristic; BNP, B-type natriuretic peptide
CKD, chronic kidney disease; AUC, area under curve; CI, confidence interval.
Supplementary Figure 2
